# Brittle bone disease (osteogenesis imperfecta): a rare condition

**DOI:** 10.11604/pamj.2022.43.11.35300

**Published:** 2022-09-07

**Authors:** Prasad Pramod Dhage, Om Chandrakant Wadhokar

**Affiliations:** 1 Department of Community Health Physiotherapy, Ravi Nair Physiotherapy College, Datta Meghe Institute of Medical Sciences, Sawangi (M), Wardha, Maharashtra, India,; 2 Department of Musculoskeletal Physiotherapy, Ravi Nair Physiotherapy College, Datta Meghe Institute of Medical Sciences, Sawangi (M), Wardha, Maharashtra, India

**Keywords:** Brittle bone, osteogenesis imperfecta, physical rehabilitation, genetic disorder

## Image in medicine

Osteogenesis imperfecta is a rare genetic disorder also known as a brittle bone disease with a prevalence of 1 in 10,000. The common clinical features of the disease include hearing loss, blue sclera, white eyes, and short stature. Other severe complications may include pulmonary valve insufficiency. The main causative agent for the condition is poorly formed by type I collagen fiber which is due to the mutation of COL1A1 or COL1A2 genes. This mutation may be hereditary. The diagnosis is usually done by radiological findings, other conditions having similar clinical findings are rickets, osteomalacia, and idiopathic juvenile osteoporosis. The available treatment strategies consist of maintaining a healthy lifestyle, avoiding smoking, high-impact activities, and a well-structured physical therapy regimen. Here we present an 11-year-old boy who came to the orthopedic department with a complaint of pain with an abnormal curve in the lower limb (A, B). As mentioned by the mother the natal history was normal with no complications, the baby was born with full-term normal delivery. The fracture at the distal third tibia (C), which is then corrected by open reduction and internal fixation, (D) shows the postoperative X-ray of the individual. After surgery, the individual was started with a physical therapy session which includes gradual strengthening of the musculature, patient and parent education regarding the condition, and joint protection techniques were taught, and eventually, ambulation was initiated.

**Figure 1:**
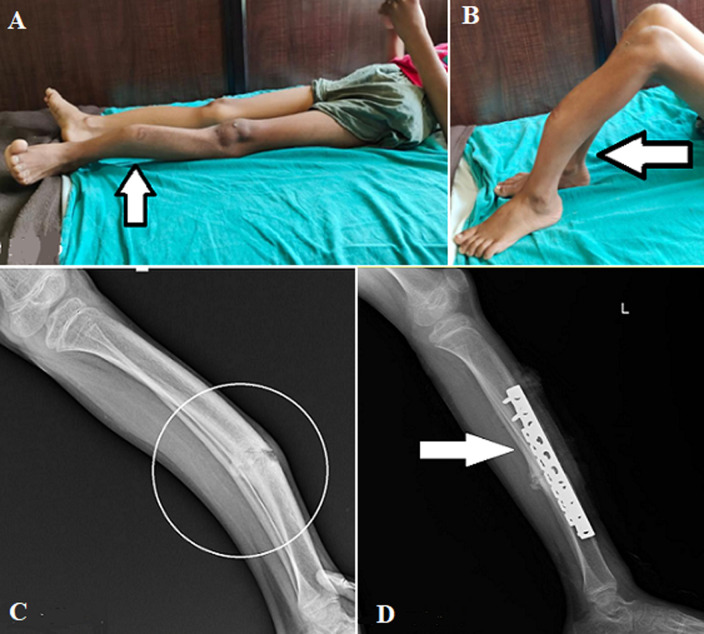
A) angular curvature of the tibia and fibula left side; B) left lower limb (tibia and fibula) showing osteogenesis imperfecta; C) angular curvature of the tibia (pre-operative); D) plating fixation of the tibia (post-operative)

